# Transforming Care Models in Cystic Fibrosis: A Review

**DOI:** 10.3390/healthcare13233022

**Published:** 2025-11-22

**Authors:** Barry Lawrence Diener, Maria Berdella, Joan DeCelie-Germana, Teresa Stables-Carney, Catherine Kier

**Affiliations:** 1 Division of Pediatric Pulmonary, Stony Brook Children’s Hospital, Renaissance School of Medicine, Stony Brook University, Stony Brook, NY 11794, USA; barry.diener@stonybrookmedicine.edu (B.L.D.); teresa.stables-carney@stonybrookmedicine.edu (T.S.-C.); 2 Division of Pulmonary and Division of Pediatrics, Lenox Hill Hospital Northwell, New York, NY 10075, USA; mberdella@northwell.edu; 3 Division of Pediatric Pulmonology, The Steven and Alexandra Cohen Children’s Medical Center, Donald and Barbara Zucker School of Medicine at Hofstra/Northwell, Lake Success, NY 11042, USA; jgermana@northwell.edu

**Keywords:** cystic fibrosis, model of care, telehealth, shared decision-making, coproduction

## Abstract

Cystic fibrosis (CF) is a multisystemic, chronic disease that requires a large multidisciplinary team for effective treatment. Over the past 20 years, the landscape of cystic fibrosis care has evolved from an almost exclusively pediatric disease to both a pediatric and adult condition. The median age of cystic fibrosis patients is rising, and the number of adults with CF is also increasing. With new developments in cystic fibrosis care, patients’ health and needs have changed, and therefore the care model of the cystic fibrosis team has also changed. The introduction of highly effective CFTR modulator therapy, the COVID-19 pandemic, and the partnership of people with CF (PwCF) and their families have catalyzed the transformation of the CF care model, which includes the growth and evolution of the CF care team given the changes in the demographics of CF patients and the incorporation of telehealth and remote patient monitoring, shared decision-making, and coproduction of care. This narrative review, focusing on the United States (US) experience, explores the transformation of CF care, highlighting demographic changes, medical breakthroughs, and systemic adaptations.

## 1. Introduction

Cystic fibrosis (CF) is an autosomal recessive disorder caused by mutations in the Cystic Fibrosis Transmembrane Conductance Regulator (CFTR) protein, leading to multisystem manifestations and respiratory failure [1]. There are over 2000 variants of the gene, and lung disease remains the principal cause of morbidity and mortality [2]. The CF care model has evolved significantly over the past two decades with the establishment of multidisciplinary teams and comprehensive care networks that have greatly improved patient outcomes [3].

Compared to 20 years ago, people with CF (PwCF) are healthier with increased survival rates [4,5,6]. When CF was first described in 1938, infants typically died within their first year of life due to bronchitis, bronchiectasis, or bronchopneumonia. Malnutrition also contributed to early deaths. [7]. While CF was traditionally seen as a pediatric disease, 58.3% of patients are now over 18 years old, with adult PwCF increasing as the pediatric population remains stable [8]. As the population ages, CF has transitioned from a childhood disease to one through the lifespan, with patients experiencing adult-onset diseases. This shift has created subpopulations, including those eligible for highly effective CFTR modulator therapy (HEMT), those with advanced CF lung disease, and those with milder CFTR dysfunction.

Current and future care delivery models must adapt to the evolving CF population. This is highlighted by the COVID-19 pandemic’s impact on the delivery of patient care in the medical field. To ensure patient and provider safety during the COVID-19 pandemic, strict infection control measures led to a surge in telehealth, with utilization increasing 50% in early 2020 compared to early 2019, and a rise of 154% by week 13 in 2020 during social shutdown orders across the United States (US) [9]. Telehealth quickly became integral to CF care, with a mean of 1.9 visits per year recorded in 2020 by the CF Foundation [10]. Remote monitoring also advanced, including home spirometry, with over 13,000 devices distributed by October 2020 and 19,000 devices by May 2021. Height, weight, and pulse oximetry monitoring were integrated into telehealth care [11]. The continued use and growth of these healthcare technologies can allow for a hybrid model of CF care with a mixing of telehealth and in-person encounters.

The CF care model must include participation by PwCF and their families. Shared decision-making increases participant knowledge, allowing for improved patient confidence and active involvement. Patients are equally or more satisfied with their decision-making process and report a positive impact on the patient-clinician communication [12]. For PwCF, a chronic, lifelong disease, this approach is crucial due to their significant treatment burden, averaging 10 daily treatments [13]. Recent advancements, such as highly effective modulator therapies (HEMT) targeting the CFTR protein, have created the need to transform CF care [14], incorporating coproduction and a shared decision-making model leading to better care for PwCF.

This current review focuses on the growth and evolution of the CF care team given the changing demographics of the CF population and the incorporation of shared decision-making and collaborative care in treating the patient with CF as a whole individual. We focus on the experience in the US and understand there are differences compared to elsewhere in the world, including in Europe, South America, and developing countries. The CF care model described in this narrative review reflects primarily the US experience, whereas European and other international models may vary in their organization and funding structures.

## 2. Growth of the Cystic Fibrosis Care Team

CF was first described in 1938 [15], and the CF Foundation (CFF) was founded in 1955 in the US as a grassroots organization by parents aiming to improve the lives of children with CF. CFF established its Care Center Network in 1961 with 2 centers [16]. By 1980, there were 115 care centers in the Care Center Network with accreditation every 5 years. In 1997, accreditation criteria were updated to include adult care in age-appropriate settings [17]. Today, the CFF remains a non-profit organization dedicating over USD 250 million annually to research for drug development, clinical care, education, and advocacy. [18]. Multidisciplinary CF care plays a critical role in the health of PwCF and their adherence to complex treatment plans. The CF care team traditionally consists of a CF physician (specializing in pediatric or adult pulmonology), a CF nurse, advanced practice providers, registered dietician, social worker, respiratory therapist, and a program coordinator. As the care of CF patients continues to become more complex, additional team members, such as genetic counselors and pharmacists, need to be included. Coordination with subspecialists in areas such as otolaryngology, gastroenterology, endocrinology, and others is critical to coordinate CF-specific care. Recent updates focus on creating a medical home with essential partners and trained and trusted referrals with expertise in CF [19]. Mental health specialists, such as psychologists and licensed clinical social workers, have been formally integrated into US CF care centers following the 2016 CFF Mental Health Guidelines [20]. This inclusion reflects the importance of addressing anxiety, depression, and adherence within comprehensive CF management.

### 2.1. Medications and the CF Pharmacist

People with CF have an immense treatment burden, often requiring on average 7 medications in their regimen [21]. This polypharmacy can lead to notable side effects. For instance, Pancreatic Enzyme Replacement Therapy (PERT) may cause stomach pain, nausea, and headaches; antibiotics can lead to allergic reactions; aminoglycosides may cause nephrotoxicity or ototoxicity; and inhaled medications may lead to bronchospasm, causing cough, dyspnea, or chest tightness. While CFTR modulators generally have a favorable safety profile, they can be hepatotoxic and cause multiple drug-to-drug interactions (DDI) [22].

A dedicated CF pharmacist can enhance care by improving discharge planning, reducing medication errors, optimizing medication usage monitoring therapies, and lowering cost [23]. Poor medication adherence has been linked to increased CF-related hospitalizations and higher healthcare costs [24]. Integrating pharmacists into CF care teams improves education of patients and staff, addresses DDI, and enhances medication access and adherence [25,26], making them a key component of CF care and a recommended part of the core team [19].

### 2.2. Anxiety and Depression

Anxiety and depression are common among PwCF [27]. A large, multicenter, multinational study assessing depression and anxiety in CF patients and their families, reported depressive symptoms in 10% of adolescents and 19% of adults and anxiety seen in 22% and 32%, respectively [28]. A more recent meta-analysis showed depression rates of 17.8–21.9% in children 5–11 years old, 19% in adolescents, and 27% in adults with CF, while anxiety was reported in 26% of adolescents and 28% of adults with CF [29]. Depression in PwCF is linked to lower quality of life (QOL), particularly in emotional functioning, eating disturbances, and body image [30], while anxiety impacts vitality, emotional functioning, social relationships, treatment burden, health perception, and respiratory symptoms [31].

To address these health concerns, the International Committee on Mental Health in Cystic Fibrosis developed guidelines recommending clinical evaluations starting at age 7, routine screening for anxiety and depression starting at 12 years old, and caregiver assessments [20]. It is also suggested that care teams identify team members who will aid in the initiation, coordination, and monitoring of treatment [20]. This led to grants from the CFF in support of the addition of a mental health coordinator to the CF care team in 2018. A mental health coordinator may be fulfilled by a clinical social worker, therapist, or psychiatrist to facilitate the implementation of these recommendations [32]. Mental health services may be provided directly by the care team or referred to external providers, depending on the resources of the individual care center. Today, mental health coordinators are an integral part of the core care team [19].

The landscape of CF mental health outcomes has changed substantially since the introduction of highly effective modulator therapy (HEMT). While earlier research identified depression rates of about 25% and anxiety rates of about 30%, emerging evidence suggests that the emotional impact of HEMT may vary in that some individuals report improved mood and hopefulness, whereas others experience new adjustment challenges related to altered disease identity or expectations of wellness [33,34]. Recent studies have begun quantifying these effects with reports of no significant reductions in depression and anxiety scores after initiation of elexacaftor/tezacaftor/ivacaftor (ETI) [35,36].

### 2.3. Palliative Care

CF presents unique palliative care opportunities and challenges due to its unpredictable course, limited life expectancy, lifelong nature, peer isolation, and potential need for lung transplantation [37]. The survey shows that over 72% of adults with CF report physical symptom burdens and 66% psychological needs [38], including pain, fatigue, difficulty sleeping, shortness of breath, fears about worsening CF, and anxiety [39]. Patients also face challenges with treatment complexity, emotional support, and decision-making [37]. Even in the era of HEMT, symptom burden continues to be a serious problem, with emotional symptoms, communication, and practical concerns often being a greater source of this burden than physical symptoms in adults with CF [40].

To better address CF patients’ palliative care needs, the CFF published 2021 guidelines recommending primary palliative care throughout the disease course by CF teams. This initiative includes palliative care education, needs screening, and comprehensive compassionate care [41]. Incorporating palliative care by the CF Care team, termed primary palliative care, into CF outpatient visits has been found to be both feasible and well-received [42]. Following training in primary palliative care, CF care teams continue to refer patients to external providers when appropriate.

### 2.4. Further Initiatives

As the needs of CF patients evolve, so does the composition of the CF care team. In addition to pharmacists, mental health coordinators, and palliative care specialists, other providers have joined the CF team through the efforts of the CFF. The Program for Adult Care Excellence (PACE) was formed to expand the scope of adult care programs and help train adult care providers [43]. The Developing Innovative Gastroenterology Specialty Training (DIGEST) program was created to help train gastrointestinal specialists on CF care [44]. Similarly, the EnVision CF: Emerging Leaders in CF Endocrinology award supports physician training with expertise in the fields of endocrinology and reproductive care [45]. The physical therapist award was designed to promote the role of physical therapy within CF care and to screen for, prevent, and treat musculoskeletal and cardiorespiratory impairments [46]. The Leadership and Education Advanced Practice Provider Fellowship Program (LEAPP) has been established to recruit and educate advanced practice providers in CF care for outpatient pediatric and adult programs [47].

## 3. The Changing Cystic Fibrosis Population and Unique Groups Within Cystic Fibrosis Care

With intensive follow-up and treatment, most children with CF now survive to adulthood [5], with over half of PwCF over the age of 18 [10]. Newborn screening has supplanted the diagnosis based on clinical manifestations, leading to improved nutritional status, respiratory status, and survival [48,49]. Earlier treatment with pancreatic enzymes, a high-calorie diet, airway clearance, chest physiotherapy, and antibiotics improved nutrition and lung function [50,51]. The increased availability of HEMT in the past decade has further enhanced survival [8]. Lung transplantation also conferred a survival benefit for adults with CF, with a greater benefit for those with more advanced disease [52]. As PwCF age, care complexity increases with complications such as hemoptysis, pneumothorax, and advanced lung disease [53,54,55,56]. Older patients are also at higher risk for CF-related diabetes mellitus, osteoporosis [1], depression, anxiety [28], and long-term antibiotic side effects like nephrotoxicity and ototoxicity [57]. They may also develop more adult diseases like cardiovascular disease [58] and cancer [59,60]. CF care providers will need to recognize the increasing rate of “new” comorbidities not commonly seen in the CF population and familiarize themselves with unique subpopulations within the CF care center, including patients on HEMT, with advanced lung disease and various CFTR-related dysfunctions. Some patients may benefit from fewer in-person visits, while others may need more frequent in-person assessments [61].

### 3.1. Effects of Highly Effective CFTR Modulator Therapy

CFTR modulators improve CFTR chloride channel function [14]. High-throughput screening has identified chemical compounds that function as potentiators, which increase the CFTR channel opening, and correctors, which act as chaperones and permit trafficking of misfolded CFTR proteins to the cell surface [62]. (Table 1) Ivacaftor (IVA), a potentiator and the first CFTR modulator, was approved for patients with the G551D gating variant in 2012, and increased lung function by 10.6% with a decrease in pulmonary exacerbations by 55% compared to placebo. It also showed a significant improvement in respiratory symptoms and weight gain, and a decrease in sweat chloride concentration [63]. The approval of IVA was the start of CFTR modulator therapy for CF and became the benchmark to which future modulators are compared. Lumacaftor-Ivacaftor (LUM/IVA), which added a corrector, LUM, to IVA approved in 2015 for Phe508del homozygous patients, showed more modest improvement in lung function and a 30–39% decrease in pulmonary exacerbations. It did not yield differences in mean body mass index (BMI), and only nominal differences in respiratory symptom scores [64]. Tezacaftor-Ivacaftor (TEZ/IVA), approved in 2018, combined TEZ, a corrector, with IVA, and demonstrated a 6.8% improvement in lung function and a 35% reduction in exacerbation in PwCF over the age of 12 years old and homozygous for Phe508del. Respiratory symptom scores also improved in the TEZ/IVA group [65]. For those over 12 years old who were heterozygous for Phe508del and a residual function variant, TEZ/IVA showed noticeable improvement in pulmonary function and respiratory symptom scores and a greater improvement than IVA alone [66].

Elexacaftor–Tezacaftor–Ivacaftor (ETI), approved in 2019, added Elexacaftor to TEZ/IVA, resulting in a 13.8% increase in lung function and a 63% reduction in exacerbations in patients at least 12 years old with one copy of Phe508del. Improvements in lung function were greater than in original trials for IVA [67]. With all HEMT, the US Food and Drug Administration (FDA) approved indications have expanded to CF patients of younger age and more eligible CFTR variants. IVA is now approved for CF patients as young as 1 month old; eligible CFTR variants for IVA increased from 1 to almost 100 variants [68]. LUM/IVA is only indicated for Phe508del homozygous individuals but is now approved for those as young as 1 year old [69]. TEZ/IVA is now approved down to age 6 years and for over 150 CFTR variants [14]. ETI is now approved for patients 2 years old and older who are carrying at least 1 copy of Phe508del or for the approved 177 ETI-responsive variants [70]. With these expanded approvals, approximately 90% of PwCF are genetically eligible for modulators [71]. Clinical benefit varies considerably across age groups. Studies demonstrate the most pronounced improvements in pulmonary function and weight in adults and adolescents [72], whereas younger children may show smaller but meaningful gains in lung function and growth [73]. In North America, before ETI became available for a given genotype or age, LUM/IVA served as an interim option for eligible homozygous F508del patients. With broad ETI eligibility, most centers now transition those patients to ETI due to superior efficacy and tolerability [72].

Vanzacaftor–Tezacaftor–Deutivactor (VTD) was approved in December 2024 for PwCF ages 6 and older who have ETI-eligible variants or one of 31 other rare mutations that were not previously approved for a modulator. VTD showed improvements in lung function comparable to ETI and reductions in sweat chloride values above those found in ETI [74,75]. VTD also has the added benefit of once per day therapy.

HEMT is generally well tolerated, but liver-function abnormalities have emerged as a key concern; severe hepatotoxicity, though rare, has led to isolated reports of liver transplantation. Current guidance recommends periodic hepatic monitoring and treatment discontinuation for marked enzyme elevations [76]. Pancreatitis has emerged in some pancreatic-insufficient patients due to residual pancreatic function, a rare occurrence prior to HEMT [77]. With CFTR modulator use, there have also been reports of anxiety and depression after starting therapy, with improvement in mood on discontinuing therapy. This was seen with both TEZ/IVA and ETI [78]. With ETI, mental fog and other mental status changes were also seen in a small number of individuals [79].

Data on treatment intolerance indicate that a minority of PwCF (<5%) discontinue HEMT because of side effects, underscoring overall tolerability but highlighting the need for individualized monitoring. Real-world and long-term extension data show that for ETI, permanent discontinuation due to adverse events occurs in approximately 1–3% of treated individuals [78,80].

Since CFTR modulators have only been approved for just over 10 years, long-term studies are needed to look at the long-term effects of HEMT on survival. Using US CF registry data, treatment with LUM/IVA predicted an increase in median survival by 6.1 years [81]. In the early IVA trial over approximately 24 weeks (6 months), adults achieved an absolute increase in percent-predicted FEV1 of approximately 5.0% (95% CI 1.15–8.78) versus placebo [82]. Real-world data for ETI, report mean improvements of 9.8% (95% CI 8.8–10.8) in the first 6 months [83], adding further hope for patients on HEMT.

Over 90% of Caucasian patients in the US have CFTR variants eligible for modulators, less so amongst patient populations: African American, Hispanic, and other racial minorities, 70%, 75%, and 80%, respectively [71]. To account for those not eligible for current HEMT, other therapeutic approaches are being evaluated.

### 3.2. Advanced CF Lung Disease

Advanced CF lung disease (ACFLD) is common, with about 18% of PwCF at 30 years old and 25% of PwCF at 45 years old having FEV1 less than 40% predicted [56]. Despite improvements in survival and QOL, it remains the leading cause of death in CF patients. HEMT and advances in CF may alter ACFLD prevalence. In 2020, the CF Foundation guidelines defined ACFLD as FEV1 < 40% predicted when “stable” (not having a pulmonary exacerbation), or referred for lung transplantation evaluation, or additional criteria such as previous intensive care unit admission for respiratory failure, hypercarbia, daytime oxygen requirement at rest, pulmonary hypertension, severe functional impairment from respiratory disease (New York Heart Association Class IV), and six minute walk test distance < 400 m. Poorer prognosis factors include frequent pulmonary exacerbations, rapid decline of forced expiratory volume in one second (FEV1), supplemental oxygen with exercise or sleep, worsening malnutrition despite supplementation, infection with difficult to manage organisms, CF-related diabetes, pneumothorax, and massive hemoptysis requiring intensive care unit admission or bronchial artery embolization [56]. CF patients with ACFLD have increased pulmonary exacerbations, higher risk of malnutrition, and are a higher risk of intensive care unit admission [84]. HEMT improves lung function and nutrition in those with ACFLD, leading to reduced lung transplantation rates [85]. Unfortunately, approximately 10% of the CF population remains ineligible for HEMT based on their genotype [10] and may be more likely to progress to ACFLD. While lung transplantation can be a key life-extending treatment, barriers include late referral, non-referral, candidacy issues, and waitlist mortality [86]. Despite advances in care and access to lung transplantation, there remains a significant increase in the likelihood of death when FEV1 has fallen below 30% predicted [87]. Patients with already established advanced lung disease and those at potentially higher risk, like PwCF not eligible for HEMT or unable to tolerate HEMT, make up a subpopulation of patients with unique challenges and needs often requiring additional discussions regarding their disease and potential for lung transplantation.

### 3.3. CFSPID/CRMS and CFTR-Related Disorders

Along the clinical spectrum of CFTR dysfunction are patients with positive newborn screens for CF and questionable diagnoses, and patients with CFTR-related disorders. When a diagnosis is uncertain, patients may eventually be reclassified as having CF, causing uncertainty for patients, families, and health professionals. Infants with positive newborn screens but who do not fit the criteria for CF are diagnosed with cystic fibrosis screen positive, inconclusive diagnosis (CFSPID, also termed cystic fibrosis transmembrane conductance regulator-related metabolic syndrome (CRMS) in the US). This applies to an asymptomatic infant with a positive newborn screen and either a sweat chloride value <30 mmol/L and two CFTR variants with at least one variant of unclear phenotypic consequences, or an intermediate sweat chloride value (30–59 mmol/L) and one or no CF causing variants [88]. With new CF newborn screen guidelines, which are designed to improve detection of PwCF and other abnormalities of CFTR function with an increased focus on CFTR genetics [89], this population is most likely to further increase. Often, interpretation of these genetics is complex, highlighting the importance of genetic counselors as core members of the CF care team [19]. These patients typically show normal growth and lung function [90]. Compared to patients with CF, patients with CFSPID/CRMS had lower initial immunoreactive trypsinogen (IRT) and sweat chloride values, better nutrition, fewer respiratory symptoms, milder or no lung disease, and less treatment burden [91,92,93]. Conversion rates or reclassification from CFSPID/CRMS to a diagnosis of CF vary from 5 to 48% [88,93]. The uncertainty of diagnosis can lead to increased parental healthcare-related uncertainty [94] and parental distress [95]. New guidelines were recently published by the CF Foundation to help guide monitoring and treatments of this unique and growing patient population [96].

CFTR-related disorders involve CFTR dysfunction but do not fully meet criteria for CF. They include congenital bilateral absence of the vas deferens (CBAVD), acute recurrent or chronic pancreatitis, bronchiectasis, and chronic rhinosinusitis. CBAVD occurs in 3% of cases of male infertility, often with one CF variant [97], commonly the splice variant IVS8-5T allele [98]. About 30% of patients with idiopathic chronic pancreatitis or recurrent acute pancreatitis have CFTR variants, and CFTR pathologic variants are found in 10–50% of bronchiectasis patients [98]. Patients with CFSPID/CRMS and CFTR-related disorders and their families face unique diagnostic and prognostic challenges, often requiring support like PwCF. With improved CFTR variant detection, previously underdiagnosed individuals can now be identified. Recognition of the role of CFTR dysfunction across multiple specialties will likely become more prevalent and require ongoing expertise of the CF provider to follow such patients long-term to establish a more definitive diagnosis.

Genetic counseling plays a central role in interpreting inconclusive CFTR variants, assessing parental carrier status, and guiding reproductive decision-making. Families benefit from counseling about recurrence risks in future pregnancies even when a classic CF diagnosis is not established [99,100]. The integration of genetic counselors within CF programs has improved patient understanding of variant interpretation, prenatal options, and newborn-screening follow-up [101].

## 4. Changing CF Care Through the COVID-19 Pandemic

The SARS-CoV-2 (COVID-19) pandemic resulted in public health measures including physical distancing, stay-at-home orders, and barriers to usual care. From 1 March 2020–31 May 2022, 42 states mandated stay-at home orders, affecting 73% of U.S. counties [102]. To prevent the spread of COVID-19, most CF Centers closed outpatient operations, and pulmonary function testing, and sputum collection were also suspended due to the risk of aerosol-generating procedures [103]. PwCF were considered at higher risk for severe outcomes due to chronic lung disease and the greater impact of respiratory viruses typically seen in CF [104,105]. This led to the rapid uptake of telehealth in CF Care Centers.

### Telemedicine and Remote Monitoring

Telehealth and telemedicine are often used interchangeably. Telehealth refers to electronic communication to improve health, including video, phone, email, remote monitoring, wearable technology, mobile applications, web portals, and even games [106]. According to the American Telemedicine Association, telehealth can include virtual visits with synchronous live, interactive encounters between a patient and healthcare provider using video, telephone, or live chat; asynchronous chat-based interaction online or via mobile application; remote patient monitoring with the use of wireless devices, wearable sensors, implanted health monitors, smartphones, and mobile applications where information is collected and transmitted for disease management; and the use of telehealth and virtual care for physician-to-physician consultation, patient education, data transmission, and digital therapeutics [107].

To maintain as close to regular care as possible and adherence to CF guidelines, routine lung function assessments and assessments of airway microorganisms, CF care teams rapidly transitioned to telehealth during the COVID-19 pandemic in the US and around the world. In the US, this shift was supported by many regulatory and payment changes to ensure patients received the care they needed [108]. While telehealth had previously been used in small studies for exercise and psychological interventions [109,110,111], its use expanded during the pandemic to circumvent pandemic- related barriers. Centers implemented both non-synchronous team-based approaches [112,113] (where care members may not always interact with patients and families at the same time) and/or synchronous [114,115].

Telehealth limitations include infrastructure development for providers and patients, reimbursement, security concerns, unequal access to technology and high-speed internet, challenges to teaching telehealth platform use, and limited interpreter services [116]. Nevertheless, telehealth adoption was positive overall, with CF patients and families expressing satisfaction and a desire to continue using telemedicine even after in-person visits resumed [117]. A large survey of PwCF and caregivers found telehealth visits to be highly convenient and effective at addressing concerns [118]. The effect on clinical outcomes is less clear. Some studies show a decrease in lung function in pediatric PwCF [119,120], while others report improvement in lung function and decreases in exacerbations and hospital admissions in pediatric and adult PwCF [121,122,123]. Overall, integration of telehealth provided access to care without compromising the health of PwCF and clinical outcomes [124]. These studies are all limited by the near simultaneous approval of ETI and pandemic-related social isolation measures.

In April 2020, the CFF began distributing home spirometers, reaching over 13,000 CF patients by October 2020, giving access to approximately 50% of patients [11]. Due to the COVID-19 pandemic restrictions, in-person lung function testing was limited. In place of in-person pulmonary function testing, spirometry coaching via virtual visits was used to improve spirometry performance. Results were found to be overall reliable for office-based spirometry [125] but may show a slight difference [126] and more variability [127]. Home spirometry may be more useful to monitor lung function stability and identify trends in lung function than absolute values [128]. Limitations include missing equipment, uncharged devices, and poor internet connection [125]. Additionally, about 90% of patients had access to a home scale, almost 50% of patients had access to a pulse oximeter, and a smaller number were able to collect home sputum, often hindered by logistic barriers [11]. Remote patient monitoring has expanded alongside telehealth, and integration of these tools within CF care networks enables more frequent assessment of lung function and adherence, especially for patients living far from accredited centers.

**Figure 1:**
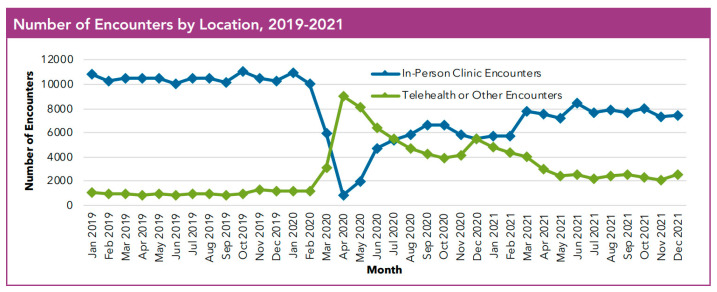
At the onset of the COVID-19 pandemic, there was a rapid increase in the use of telehealth encounters at US CF Centers with the continued use of telehealth alongside in-person encounters [10]. Reproduced with permission from CFF, 2023. Source of data: Cystic fibrosis patients under care at CF Foundation-accredited care centers in the United States, who consented to have their data entered.

The pandemic shifted healthcare consumption in March 2020; there was a rapid increase in telehealth encounters for patients with CF. While telehealth visits decreased from their peak in April 2020, there has been continued use of telehealth in the CF community (Figure 1) [10]. In 2020, there were 57,921 telehealth encounters in the US Cystic Fibrosis Registry with persistent use but with a noted decline in the number of telehealth encounters, with 35,525 in 2021, and 25,553 in 2022 [129]. Most recently in 2024, there were 11,312 CF telehealth encounters in the US Cystic Fibrosis Registry [130]. While telehealth encounters continue to decline and many PwCF have returned to in-person care, telehealth continues to offer flexibility and an important avenue of care for PwCF. Moving forward, telehealth and remote patient monitoring will continue to play a role in CF care and need to be better defined through continued research.

## 5. Communication and Partnership

### 5.1. Shared Decision-Making

Shared decision-making is a patient centered healthcare model that puts the patient at the center of the decision-making process and ensures patients and families are engaged in decision-making about their care with the healthcare team [131]. Shared decision-making accepts that individual self-determination is a desirable goal that needs to be supported by the clinical care team. Patients and families need to be informed and should not make decisions about key issues with insufficient information. They need to be supported during their decision-making process [132].

The shared decision-making model has been previously evaluated in various other chronic diseases, including allergy immunotherapy [133], inflammatory bowel disease [134], head and neck surgery [135], mental illness [136], and cancer care [137]. It has been shown to improve satisfaction, increase knowledge, and reduce decisional conflict [138]. Open communication between the CF care team, PwCF, and families is a crucial part of CF care. In a large study assessing the use of shared decision-making in CF care, 69% of respondents reported some experience with shared decision-making [139].

**Figure 2:**
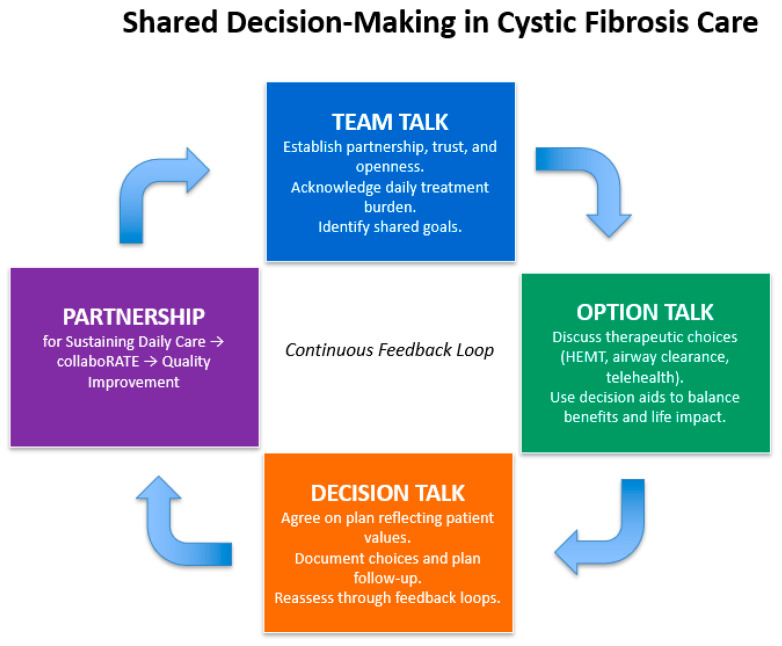
A simplified decision aid to highlight the components of shared decision-making, adapted from Elwyn et al. (2012) [132], Homa et al. (2021) [139], and CF Foundation Partnerships for Sustaining Daily Care (2016) [142].

Decision aids support the shared decision-making process by providing patients with tailored information. For example, a tool for PwCF was developed using an analytical hierarchy approach to evaluate treatment based on patient goals such as preventing lung infection, improving breathing function, improving feelings of well-being, and minimizing treatment burden and cost. Treatments were analyzed using an analytical hierarchy approach to develop a tool for the effectiveness of treatments. These tools enhance engagement through collaboration and individualized care [140]. Another tool was used for patients with end-stage CF lung disease referred for lung transplantation, resulting in improved knowledge, more realistic expectations about risk and survival, and reduced decision conflict compared to usual care and counseling [141]. A simplified decision aid to highlight the components of shared decision-making could be found in Figure 2. 

### 5.2. Coproduction

Coproduction involves partnering patients with their care team to set agendas and mutual goals of care. Agenda setting minimizes unexpected topics without increasing visit length or reducing the number or importance of topics discussed [143]. PwCF values collaborative interactions with their healthcare team and wants their team to listen and respond effectively. Goal setting tools and training are recommended to help clinicians, PwCF, and families prepare for conversations [144].

Beyond the clinical setting, coproduction has played a key role in CF guideline development. Since 2004, the CF Foundation has included patients and/or families in the development of CF care guidelines, with greater involvement seen in the 2018 CFTR modulator guidelines. CF patient and family participation is overwhelmingly positive, improving the guideline formation process. Patient involvement in open public forums, surveys, focus groups, and committees has enhanced the relevance and quality of guidelines. Clinicians, patients, and family members add that the lived experience is an essential aspect in the creation of guidelines [145]. PwCF and their families can also partner on quality improvement (QI) initiatives, offering unique perspectives. As QI leads to improved care, coproduced QI initiatives motivate patients and families with a shared aim [146]. Many U.S. CF care centers include patient and family advisory boards, moderated and driven by patients to approach challenges and celebrate accomplishments within a center; this also opens QI opportunities to improve overall patient satisfaction in their care. In one study, PwCF partnered with the clinicians to develop a dashboard that displayed patient-reported and clinical data, enabling a more patient-centered approach and prioritization of concerns during visits [147]. Partnering with PwCF and their families placed them at the center of care, promoting shared decisions for individualized care. While positive feedback on shared decision-making in promoting collaborative care exists, further studies are needed to evaluate short- and long-term outcomes. Furthermore, engaging PwCF and families offers valuable insight into their unique experiences and perspectives, informing guideline formation and quality improvement projects.

## 6. Limitations

As a narrative review, this is useful for providing an overview of the transforming care models in CF, but it has several limitations inherent to narrative reviews. They are subjective, leading to bias. Unlike systematic reviews, they lack a standardized methodology, making them less transparent and difficult to reproduce. Additionally, narrative reviews do not include quantitative analysis, so they cannot provide pooled data or assess the strength of evidence. As a result, their conclusions may be less reliable for guiding practice or policy decisions. This narrative review focuses primarily on the US experience and does not comprehensively analyze international healthcare system structures. Although the literature was searched comprehensively, it is possible that recent studies were missing. Future research should compare international models and evaluate long-term outcomes of telehealth, mental health integration, and coproduction initiatives. Continued collaboration among clinicians, researchers, and PwCF will ensure that care models remain responsive, equitable, and evidence based.

## 7. Conclusions

Since first being described, our knowledge and understanding of CF has significantly advanced, leading to better treatments and improved care. This has led to large breakthroughs and individualized care highlighted by the development of CFTR modulators, which have led to improved lung function, nutrition, and QOL. PwCF are living longer and healthier lives. As health needs evolve, resources may need to be reallocated, i.e., shifting focus from inpatient care to outpatient services. It is essential to consider specific subpopulations of patients, such as those with advanced lung disease, those who are not eligible for HEMT, and those with uncertain diagnoses. Further consideration and effort are also crucial to address and resolve social disparities in CF care.

CF care teams have changed through initiatives as patients needs change. Mental health coordinators, pharmacists, CF gastroenterologists and endocrinologists, respiratory therapists, physical therapists, and advanced care providers are now integral parts of the CF team [19]. How we provide care has also changed, accelerated by the COVID-19 pandemic. With the changing needs of PwCF, how and where care teams provide care will also change. Some patients whose health is stable may need fewer in-person visits [3], others may benefit from a combination of telehealth visits and remote patient monitoring, while others may need more frequent in-person assessments of their health. Telehealth and remote patient monitoring are now part of CF care, and the partnership between PwCF, families of CF patients, and the CF care team is the cornerstone of patient-centered multidisciplinary care.

With the evolution of the CF care model, there will be further changes as new research and needs are identified. Further collaboration with essential partners and CF trained subspecialists needs to be further developed [19]. As technology continues to permeate our society, further means of remote patient monitoring, home spirometry, use of personal devices and applications, patient use of the electronic medical record, and newer technology should be leveraged and embraced. Going forward, partnership with patients and families should be prioritized in and outside of the clinic to co-produce research and QI.

Further research is needed on the changing care model’s effects and how future developments will continue to change CF care.

## Figures and Tables

**Table 1:** CFTR modulator availability in the US as of 2025.

Modulator Name	Year of First US-FDA Approval	Initial CFTR Variants Approved	CFTR Mutations for Which It Is Approved	Initial Age Approval	Current Age Approval
Ivacaftor	2012	G551D	97 variants ^a^	≥6 years	≥1 month
Lumacaftor/ivacaftor	2015	Homozygous Phe508del	Homozygous Phe508del	≥12 years	≥1 years
Tezacaftor/ivacaftor	2018	Homozygous Phe508del or at least 1 of 26 additional TEZ/IVA responsive mutations	Additional 127 TEZ/IVA responsive variants ^b^	≥12 years	≥6 years
Elexacaftor/tezacaftor/ivacaftor	2019	At least 1 copy of Phe508del	Additional 272 ETI-responsive variants ^c^	≥12 years	≥2 years
Vanzacaftor/tezacaftor/deutivacaftor	2024	At least 1 copy of phe508del, OR ETI responsive variants OR 31 additional variants ^d^		≥6 years	

Abbreviations: CFTR, cystic fibrosis transmembrane conductance regulator; IVA, Ivacaftor; ETI, Elexacaftor plus tezacaftor and ivacaftor; TEZ/IVA, Tezacaftor plus ivacaftor; US-FDA, United States Food and Drug Administration. ^a^ All IVA responsive variants are listed on the package insert: https://pi.vrtx.com/files/uspi_ivacaftor.pdf (accessed on 19 August 2025). ^b^ All TEZ/IVA responsive variants are listed on the package insert: https://pi.vrtx.com/files/uspi_tezacaftor_ivacaftor.pdf (accessed on 19 August 2025). ^c^ All ETI responsive variants are listed on the package insert: https://pi.vrtx.com/files/uspi_elexacaftor_tezacaftor_ivacaftor.pdf (accessed on 19 August 2025). ^d^ VTD responsive variants are listed on package insert: https://pi.vrtx.com/files/uspi_vanzacaftor_tezacaftor_deutivacaftor.pdf (accessed on19 August 2025 ).

## Data Availability

No new data were created or analyzed in this study. Data sharing is not applicable to this article.

## Abbreviations

The following abbreviations are used in this manuscript:

CF	Cystic fibrosis
PwCF	People with CF
CFTR	cystic fibrosis transmembrane conductance regulator
HEMT	highly effective CFTR modulator therapy
